# Childhood glaucoma profile in a Southwestern Ethiopia tertiary care center: a retrospective study

**DOI:** 10.1186/s12886-023-03268-7

**Published:** 2024-01-22

**Authors:** Tarekegn Mulugeta, Guteta Gebremichael, Sufa Adugna

**Affiliations:** Department of Ophthalmology, Institute of Health, Jimma University, Jimma, Ethiopia

**Keywords:** Childhood glaucoma, Primary congenital glaucoma, Childhood blindness

## Abstract

**Background:**

Childhood glaucoma is a major cause of childhood blindness worldwide. The profile of childhood glaucoma has not been well characterized in sub-Saharan Africa. Thus, this study was designed to describe demographics, clinical features, managements of childhood glaucoma, and improvements in visual acuity (VA) and intraocular pressure (IOP) from baseline to final visit.

**Methods:**

This retrospective study included glaucoma patients below 18 years old who were diagnosed between September 2019 to August 2022. Childhood glaucoma diagnosis and classification was made as per the Childhood Glaucoma Research Network Classification (CGRN).

**Results:**

A total of 105 children (181 eyes) were diagnosed with glaucoma. The most common type of childhood glaucoma was primary congenital glaucoma (PCG) constituting (42%, *n* = 76 eyes, 95% confidence interval (CI), 34.7–49.5%; *P* = 0.037), followed by glaucoma suspect (22.1%, *n* = 40 eyes, 95% CI, 16.3–28.9%; *P* < 0.001) and juvenile open-angle glaucoma (JOAG) (15.5%, *n* = 28 eyes, 95% CI, 10.5–21.6%; *P* < 0.001). While the most common type of secondary glaucoma was steroid-induced glaucoma, followed by glaucoma following cataract surgery. Bilateral glaucoma was found in 72.4% (*n* = 76 children, 95% CI, 62.8–80.7%; *P* < 0.001) of children. In both primary and secondary glaucoma, boys were affected more than girls, in ratio of 2:1 and 2.7:1, respectively. The mean age at presentation for patients with PCG was 2.7 years. Close to 93.4% (71) of PCG eyes were managed surgically, of which majority underwent combined trabeculotomy and trabeculectomy (CTT). Most of secondary glaucoma cases were treated medically. Overall, 85.3% (111) of eyes had successful control of IOP ≤ 21 mmHg.

**Conclusion:**

PCG was the most common type of childhood glaucoma. One of a well-recognized challenge in developing countries, late presentation of patients with PCG, was also observed in our study. Which highlights, the need of increasing access to eye-care service and awareness of childhood glaucoma as a major public health issue. Steroid-induced glaucoma was the most common type of secondary glaucoma; appropriate measures should be taken to prevent this preventable glaucoma.

## Background

Childhood glaucoma is a vision threatening condition [1, 2]. World Glaucoma Association and United States Board of Ophthalmology defined childhood glaucoma as a disease onset at < 18 years of age and is characterized by intraocular pressure (IOP)-related eye damage caused by a variety of conditions [3, 4]. It is responsible for 5% of global childhood blindness and 11% of African childhood blindness [5, 6]. Despite these very large proportions, childhood glaucoma does not receive the attention it merits. In Africa, there is generally a delay in the presentation of affected children due to illiteracy in the general population, with more severe diseases phenotypes [7]. The main factors influencing its prognosis are an early, accurate diagnosis and successful IOP reduction to a level where progression is unlikely [8]. Otherwise, due to further damage to the immature visual system, the visual effects of childhood glaucoma are frequently more severe [9].

**Fig. 1:**
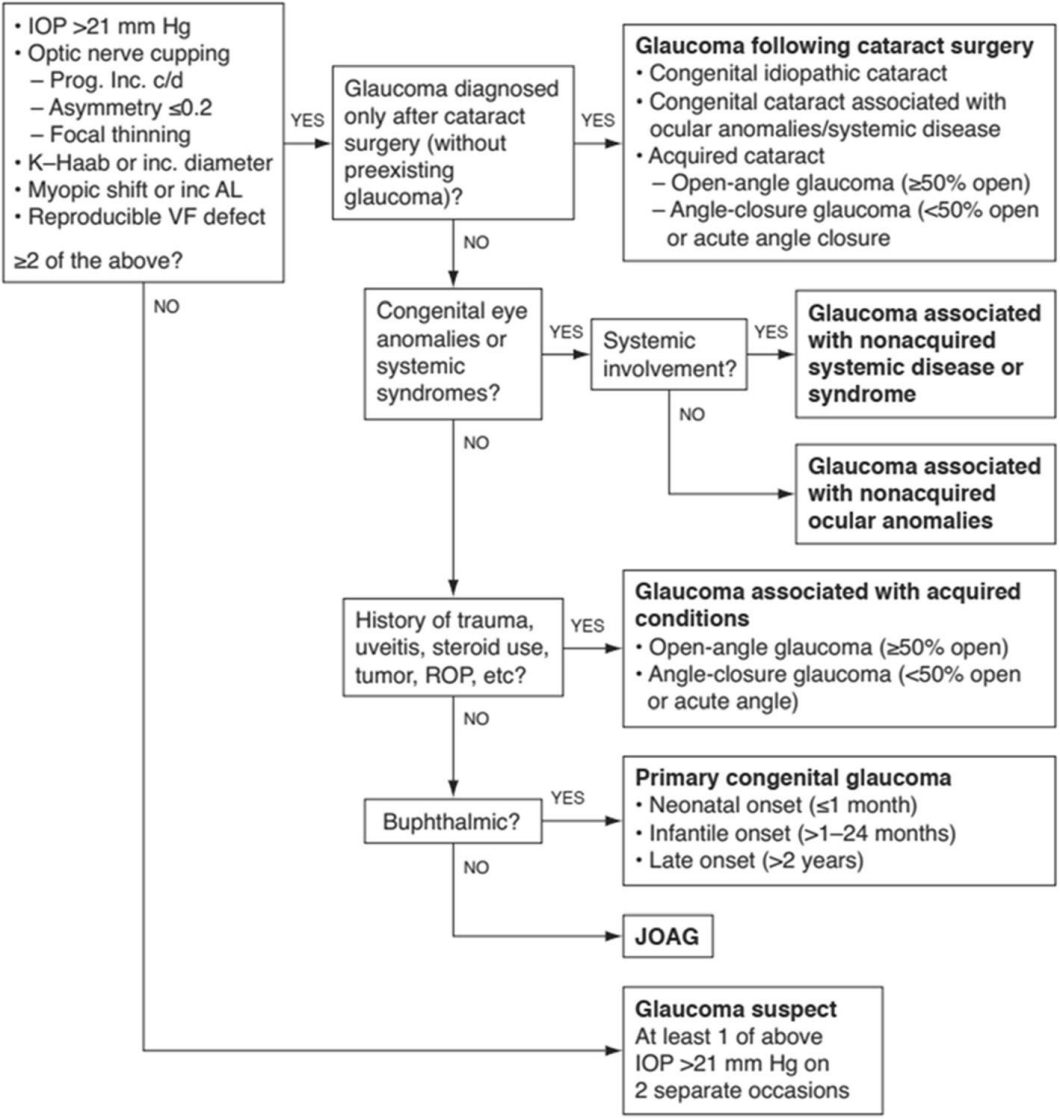
Childhood Glaucoma Research Network/World Glaucoma Association algorithm for the classification of childhood glaucoma. AL = axial length; C/D = cup-disc; JOAG = juvenile open-angle glaucoma; ROP = retinopathy of prematurity; VF = visual field [4]

According to the CGRN classification (see Fig. 1), childhood glaucoma classified into primary glaucoma, secondary glaucoma and glaucoma suspect [4]. Primary glaucoma encompasses primary congenital glaucoma (PCG) and juvenile open-angle glaucoma (JOAG), whereas secondary glaucoma is classified based on the underlying pathology. Secondary glaucoma includes glaucoma associated with nonacquired ocular anomalies (e.g., Axenfeld-Rieger spectrum, iris hypoplasia, aniridia), glaucoma associated with nonacquired systemic disease (e.g., phacomatoses, Juvenile Idiopathic Arthritis [JIA]), and glaucoma associated with acquired conditions (e.g., uveitis, trauma, or intraocular surgery). Glaucoma following cataract surgery is classified separately [6]. Understanding the patterns and associated clinical characteristics of the various glaucoma subtypes can aid in collaborative research and the development of effective treatment and management regimens.

The purpose of this study was to provide the epidemiology, the recent classification, the clinical features, and the treatment modalities instituted and future strategies to combat childhood blindness due to glaucoma in children. Furthermore, the findings of this study address the scarcity of studies on the profile of childhood glaucoma in Sub Saharan Africa based on the CGRN classification.

## Methods

We conducted a hospital-based retrospective cross-sectional study in which we reviewed and analyzed the medical records of all glaucoma patients below the age of 18 who presented to Jimma Medical Center (JMC) between September 2019 and August 2022. Medical records of patients with missing information were excluded from the study. The extracted patient data included: (1) demographics such as age at presentation, gender, consanguinity, and family history of childhood-onset glaucoma; (2) clinical data such as diagnosis according to the CGRN classification [4], laterality, visual acuity (VA) and method of testing, intraocular pressure (IOP) measurement, medications, tonometer, and details of examination under anesthesia (EUA), where applicable; and (3) management data such as medication and surgery. The best-corrected quantitative VA was converted to the equivalent logarithm of the minimum angle of resolution (logMAR) VA. If a VA assessment was not recorded, it was documented as unrecorded, and the logMAR VA was marked as missing. The following logMAR VA were assigned to certain nonquantitative visual acuities: Counting fingers = 2.10; hand motions = 2.40; light perception = 2.70; and no light perception = 3.00 [10]. All children's IOP was measured with an iCare tonometer. In young and uncooperative children IOP measured during an EUA using ketamine anesthesia. Combination eye drops were recorded as two separate medications.

Medical records with at least 6 months of follow-up data were analyzed for outcome. The definition of success was a numerical IOP ≤ 21 mmHg without (complete success) or with (qualified success) medications.

The collected data were entered into Epidata version 3.1 and extracted to SPSS version 26 for analysis. Categorical variables were summarized with numbers and percentages, while continuous variables were summarized with means and standard deviations (SDs). Differences between baseline and final visits IOP (mmHg) and VA (logMAR) were assessed using paired sample *t*-tests and analysis of variance when parametric statistics were appropriate and using paired Wilcoxon tests when nonparametric statistics were appropriate. *P* ≤ 0.05 was considered statistically significant.

The College of Medicine Research and Ethics Committee approved the study. The Institutional Review Board (IRB) determined that formal informed consent was not required because no personally identifiable information was gathered during the review of the medical records.

## Results

### Demographics

A total of 105 patients (181 eyes), were seen in Ophthalmology Department of JMC during the 3-year study period. Majority, 72.4% (*n* = 76 children, 95% CI, 62.8 – 80.7%; *P* < 0.001), of patients had bilateral ocular involvement. There were 62.9% (*n* = 66) males and 37.1% (*n* = 39) girls, with a mean age of 8.3 years at the time of diagnosis (SD, 6.6 years; median, 7 years).

### Gender and age

Males presented with PCG at a higher rate (73.8%) than females (Table 1). The mean age at presentation for PCG patients was 2.7 years, with a SD of 3.1 years (median 1.2). While, the mean age at presentation for patients with secondary glaucoma was 8.8 years, with a SD of 4.7 years (median 9.1) (Table 2).

**Table 1:** Demographic Characteristics

	**All patients**	**PCG Patients**
	*n* (%)	*n* (%)
Total	105	42
Gender		
Male	66 (62.9%)	31 (73.8%)
Female	39 (37.1%)	11 (26.2%)
Age at presentation (years)		
Mean	8.2	2.7
SD	6.4	3.1
Family history of childhood-onset glaucoma		
Yes	3 (2.9%)	-
No	92 (87.6%)	37 (88.1%)
Unknown	10 (9.5%)	5 (11.9%)
Consanguinity		
Yes	-	-
No	3 (2.9%)	3 (7.1%)
Unknown	102 (97.1%)	39 (92.9%)

*Abbreviations*: *PCG* Primary Congenital Glaucoma, n (%) number and percent of total patients, *SD* Standard deviation

**Table 2:** Clinical Characteristics of patients with various types of childhood glaucoma

	*n* (%)	Laterality (Unilateral: Bilateral)	Gender (Male: Female)	Age at presentation (years) (Mean ± SD, Median, IQR)
**Primary Childhood Glaucoma**	**104 (57.5%)**	**11:93**	**69:35**	**6.3 ± 2.5, 3.0, 13.3**
Primary Congenital Glaucoma	76 (42.0%)	7:69	55:21	2.7 ± 3.1, 1.2, 3.9
Juvenile Open-Angle Glaucoma	28 (15.5%)	4:24	14:14	16.2 ± :1.9, 17.3, 2.5
**Secondary Childhood Glaucoma**	**37 (20.4%)**	**13:24**	**27:10**	**8.8 ± 4.7, 9.1, 7.8**
Glaucoma Associated with Acquired Condition	14 (7.7%)	6:8	12:2	11.8 ± 4.9, 13.5, 5.7
Glaucoma Associated with Nonacquired Ocular Anomalies	4 (2.2%)	0:4	4:0	10.5 ± 2.9, 10.5, 5.0
Glaucoma Associated with Nonacquired Systemic Disease or Syndrome	11 (6.1%)	1:10	5:6	6.8 ± 3.3, 8.0, 7.5
Glaucoma following Cataract Surgery	8 (4.4%)	6:2	6:2	5.5 ± 3.4, 4.5, 6.8
**Glaucoma suspect**	**40 (22.1%)**	**5:35**	**14:26**	**12.4 ± 5.3, 15.0, 9.3**
**Total**	**181 eyes**	**29:152**	**110:71**	**8.2 ± 6.4, 7.0, 13.0**

Abbreviations: n (%), number and percent of total eyes, *IOP* Intraocular Pressure, *SD* Standard Deviation, *IQR* Interquartile Range, *PCG* Primary Congenital Glaucoma, *JOAG* Juvenile Open-Angle Glaucoma

### Family history of childhood-onset glaucoma and consanguinity

A family history of childhood-onset glaucoma was found in approximately 3% of all patients. However, for most patients’ the history of consanguinity was unknown (Table 1).

### Diagnosis

PCG was the most common (*n* = 76 eyes, 42.0%, 95% confidence interval (CI), 34.7—49.5%; *P* = 0.037) type childhood glaucoma, followed by glaucoma suspect (*n* = 40, 22.1%, 95% CI, 16.3 – 28.9%; *P* < 0.001) and JOAG (*n* = 28, 15.5%, 95% CI, 10.5 – 21.6%; *P* < 0.001) (Table 3). Glaucoma suspect was diagnosed in 26 (65%) eyes by suspicious optic nerve cupping and in 14 (35%) eyes by IOP > 21 mmHg. Steroid-induced glaucoma was the most common cause of secondary childhood glaucoma, accounting for 9 (5.0%) of all cases, followed by glaucoma following cataract surgery in 8 (4.4%) eyes.

**Table 3:** Diagnosis of Childhood Glaucoma

	*n* (%)	*n* (%)	*n* (%)
Primary Childhood Glaucoma	104 (57.5%)		
Primary Congenital Glaucoma		76 (42.0%)	
Juvenile Open-Angle Glaucoma		28 (15.5%)	
Secondary Childhood Glaucoma	37 (20.4%)		
Glaucoma Associated with Acquired Condition		14 (7.7%)	
Trauma			1 (0.6%)
Uveitis			1 (0.6%)
Steroid-induced			9 (5.0%)
Tumor			1 (0.6%)
Other			2 (1.1%)
Glaucoma Associated with Nonacquired Ocular Anomalies		4 (2.2%)	
Aniridia			4 (2.2%)
Glaucoma Associated with Nonacquired Systemic Disease or Syndrome		11 (6.1%)	
Phacomatoses: Sturge-Weber syndrome			3 (1.7%)
Phacomatoses: Neurofibromatosis type 1			6 (3.3%)
JIA			2 (1.1%)
Glaucoma following Cataract Surgery		8 (4.4%)	
Aphakia			2 (1.1%)
Pseudophakia			6 (3.3%)
Glaucoma suspect	40 (22.1%)		
Total	181 eyes		

Abbreviations: *PCG* Primary Congenital Glaucoma, *JOAG* Juvenile Open-Angle Glaucoma, *JIA* Juvenile Idiopathic Arthritis, *SD* Standard Deviation

### Baseline visit clinical assessment

#### Visual acuity

Baseline visit quantitative VA was recorded in 63.0% (*n* = 114 eyes) of all childhood glaucoma eyes (*n* = 181) and in only 22.4% of PCG eyes (considering age at diagnosis). The overall mean VA of the 114 eyes that had a quantitative VA at the baseline visit was 0.92 logMAR (SD, 1.06) (Table 4).

**Table 4:** Baseline and Final Visit Quantitative Visual Acuity

	**Baseline visit VA (logMAR)**			**Final visit VA (logMAR)**			**paired *t* test**		
	*n*	Mean	SD	*n*	Mean	SD	Mean logMAR VA difference	95% CI	*p*-value
All Childhood Glaucoma	114	0.92	1.06	111	0.94	1.11	0.01	-0.09—0.06	*p* = 0.745
PCG	17	1.98	0.73	18	2.22	0.72	-0.26	-0.5—-0.02	***p* = 0.033**
JOAG	28	1.31	1.13	26	1.53	1.15	-0.15	-0.36—0.07	*p* = 0.17
Secondary Glaucoma	30	1.00	1.05	28	0.78	0.94	0.18	0.01—0.35	***p* = 0.033**
Glaucoma Suspect	39	0.10	0.21	39	0.07	0.16	0.04	0.02—-0.01	*p* = 0.132

*Abbreviations*: *n* number of eyes, *IOP* Intraocular Pressure, *CI* Confidence interval, *SD* Standard Deviation, *PCG* Primary Congenital Glaucoma, *JOAG* Juvenile Open-Angle Glaucoma

### Intraocular pressure

Of 181 eyes with childhood glaucoma or glaucoma suspect IOP was recorded for 157 eyes. Of 76 eyes with PCG, 57 (75%) of them have baseline IOP measurements. For 9 (15.8%) of them the measurement was taken during an EUA under ketamine anesthesia. The mean IOP for all eyes was 34.6 ± 13.4 mmHg, and 41.3 ± 9.8 mmHg for PCG (Table 5).

**Table 5:** Baseline and Final Visit Intraocular Pressure

	**Baseline visit IOP (mmHg)**			**Final visit IOP (mmHg)**			**paired *t* test**		
	*n*	Mean	SD	*n*	Mean	SD	Mean IOP reduction	95% CI	*p*-value
All Childhood Glaucoma	157	34.6	13.4	130	16.7	6.3	20.3	17.9—22.7	***p* < 0.001**
PCG	53	41.3	9.8	57	17.0	7.7	24.5	20.7—28.2	***p* < 0.001**
JOAG	28	43.8	12.6	23	16.0	4.7	25.7	20.0—31.3	***p* < 0.001**
Secondary Glaucoma	36	32.7	11.8	31	17.0	6.0	17.1	17.0—34.1	***p* < 0.001**
Glaucoma Suspect	40	21.1	6.6	19	16.2	3.0	8.8	5.8—11.8	***p* < 0.001**

Abbreviations: *n*, number of eyes, *IOP* Intraocular pressure, *CI* Confidence interval, *SD* Standard deviation, *PCG* Primary congenital glaucoma, *JOAG* Juvenile open angle glaucoma

### Management

#### Primary congenital glaucoma

In this study PCG was the most common type of childhood glaucoma (76 of 181 eyes [42.0%]), its management analyzed in detail. Majority of PCG eyes (71/76, 93.4%) were treated surgically, and 6.6% received medical treatment only (*n* = 5 eyes) (Table 6). Among the eyes that underwent surgery, 94.4% (67/71) had anti-glaucoma medication prior to surgery, and on average, 2.04 classes of medication were prescribed (range, 1–5). The most commonly prescribed medication was a beta-blocker, followed by oral carbonic anhydrase inhibitors (CAIs), topical CAIs, and prostaglandin analogues.

### Surgery

For PCG eyes, surgery was carried out within 7 days of the baseline visit in 52.1% (*n* = 37 eyes), 14 days in 81.7% (*n* = 58 eyes), and 28 days in 90.1% (*n* = 64 eyes). Overall, it took around 18 days on average from the baseline visit to the first surgery (SD, 30.5 days; median, 7 days; IQR, 7.0 days) (Table 6).

**Table 6:** Management of Childhood Glaucoma or Glaucoma Suspect Eyes

	**All Childhood Glaucoma Eyes**		**PCG Eyes**		**JOAG Eyes**		**Secondary Childhood Glaucoma Eyes**		**Glaucoma Suspect Eyes**	
	No	%	No	%	No	%	No	%	No	%
Surgery	79	43.6	71	93.4	7	25.0	**-**	**-**	-	-
Medication/no surgery	72	39.8	5	6.6	19	67.8	36	97.3	12	30.0
No treatment	29	16.0	-	-	1	3.6	1	2.7	28	70.0
No treatment recorded	1	0.6	-	-	1	3.6	-	-	-	-
Total eyes	181		76		28		37		40	
Eyes treated with medication before surgery	74	93.7	67	94.4	7	100.0	-	-	-	-
No. of medications before surgery										
Mean	1.99		2.04		2.42		1.86		1.17	
SD	0.85		0.59		1.10		0.99		0.39	
Types of medication before surgery*										
Beta-blocker	142	97.3	72	100.0	22	84.6	36	100.0	12	100.0
Prostaglandin analogue	32	21.9	10	13.9	13	50.0	9	25.0	-	-
Alpha agonist	2	1.4	-	-	2	7.7	-	-	-	-
Topical CAI	35	24.0	10	13.9	10	38.5	15	41.7	-	-
Oral CAI	80	54.8	55	76.4	16	61.5	7	19.4	2	16.7
Days from baseline visit to first Surgery										
Up to 1 week	38	48.1	37	52.1	1	14.3	-	-	-	-
Up to 2 weeks	61	77.2	58	81.7	2	28.6	-	-	1	100.0
Up to 4 weeks	67	84.8	64	90.1	2	28.6	-	-		
Mean	28.41		17.93		137.29		-		10.00	
SD	85.57		30.53		262.90		-		-	
No. of glaucoma surgeries per eye										
1	56	70.9	48	67.6	7		-	-	1	100.00
2	21	26.6	21	29.6	-		-	-		
3	1	1.25	1	1.4	-		-	-		
4	1	1.25	1	1.4	-			-		
Mean	1.33		1.37		1.00		-			
SD	0.57		0.59		0.00		-			

*Abbreviations*: *PCG* Primary Congenital Glaucoma, *JOAG* Juvenile Open-Angle Glaucoma, *CAI* Carbonic Anhydrase Inhibitor, *SD* Standard Deviation

^*^Because some eyes received more than one type of medication prior to surgery, the percentages may exceed 100%

Of the PCG eyes that had surgery, 67.6% (*n* = 48) had only one glaucoma surgery. Of the 32.4% (*n* = 23) of eyes that had more than one glaucoma surgery, 29.6% (*n* = 21) had two operations, 1.4% (*n* = 1) had three, and 1.4% (*n* = 1) had four total operations per eye (Table 6).

**Fig. 2:**
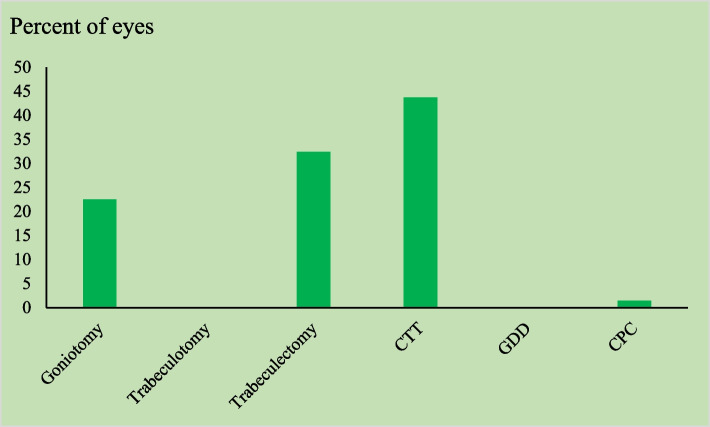
Initial Surgery for PCG eyes. CTT = Combined trabeculotomy-trabeculectomy, GDD = Glaucoma Drainage Device, CPC = trans-scleral cyclophotocoagulation

The initial surgical procedure of choice for PCG eyes that underwent surgery was combined trabeculotomy-trabeculectomy (CTT) in 43.7% (31/71 eyes), followed by trabeculectomy 32.4% (23/71), goniotomy 22.5% (16/71), and trans-scleral cyclophotocoagulation (CPC). Primary glaucoma drainage device (GDD) surgery and trabeculotomy were not the first-line surgery for any PCG eyes (Fig. 2).

**Fig. 3:**
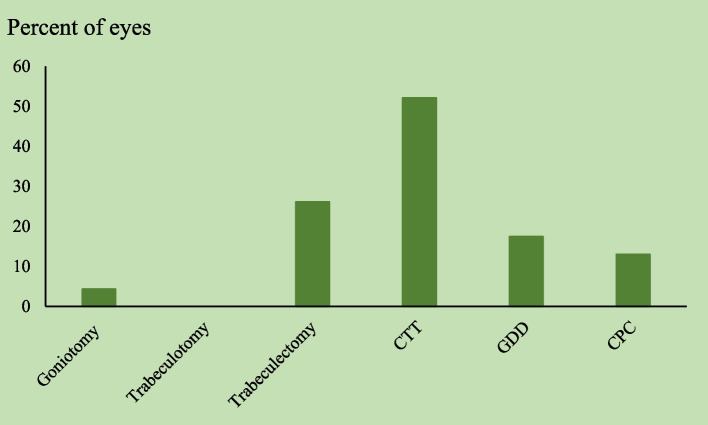
Secondary surgery for PCGs. CTT = Combined trabeculotomy-trabeculectomy, GDD = Glaucoma Drainage Device, CPC = trans-scleral cyclophotocoagulation

Of 71 PCG eyes treated surgically, 23 eyes underwent more than 1 surgery (26 procedures), most underwent CTT (*n* = 12, 52.1%), followed by trabeculectomy (*n* = 6, 26.1%), GDD (*n* = 4, 17.4%), CPC (*n* = 3, 13.0%), and goniotomy (n = 1, 4.3%) (Fig. 3).

### Glaucoma suspect

There were 40 (22.1%) eyes with glaucoma suspect, majority of them 28 (70.0% of total glaucoma suspect eyes) were managed conservatively. While 30.0% (*n* = 12) of eyes were managed with medication (Table 6).

### Secondary childhood glaucoma

Thirty-seven (20.4%) eyes had secondary childhood glaucoma, of which who had treatment record, all of them were treated medically (Table 6).

### Juvenile open-angle glaucoma

Twenty-eight (15.5%) eyes had JOAG, and 19 (67.8%) of those were treated medically. Surgery was performed in 20.0% (*n* = 7) (Table 6).

### Final visit clinical assessment

Of the 181 eyes with childhood glaucoma, 111 (61.3%) eyes had final visit quantitative VA (Table 4) and 130 (71.8%) eyes IOP records (Table 5).

### Visual acuity

Final visit quantitative VA was recorded in 61.3% (*n* = 111 eyes) of all childhood glaucoma eyes (*n* = 181) and in only 23.7% of PCG eyes (considering age at diagnosis). The overall mean VA of the 111 eyes that had a quantitative VA at the final visit was 0.94 logMAR (SD, 1.11) (Table 4). When assessing only eyes with both baseline and final visit quantitative VA, there was a non-statistically significant deterioration in mean quantitative VA from baseline compared with final visit for the group overall (mean logMAR VA difference = 0.01, 95% confidence interval [CI], -0.09–0.06; *P* = 0.745, paired *t* test). For eyes with PCG, there was statistically significant deterioration (mean logMAR VA difference = -0.26, 95% CI, -0.5—-0.02; *P* = 0.033, paired *t* test). Which could be because of inappropriate documentation or because of the nature the study design and it needs prospective study. Furthermore, for eyes with JOAG there was a non-statistically significant deterioration in mean quantitative VA (mean logMAR VA difference = -0.15, 95% CI, -0.36—0.07; *P* = 0.17, paired *t* test). For eyes with secondary glaucoma, there was statistically significant improvement (mean logMAR VA difference = 0.18, 95% CI, 0.01—0.35; *P* = 0.033, paired *t* test) while for glaucoma suspect there was a non-statistically significant improvement (mean logMAR VA difference = 0.04, 95% CI, 0.02—-0.01; *P* = 0.132, paired *t* test) (Table 4).

### Intraocular pressure

IOP was successfully controlled in 85.4% (111/130) of the eyes at the final visit, with 43.8% of complete and 41.5% of qualified success (Table 7). In contrast, 14.6% of failed cases had IOPs greater than 21 mmHg.

Of the 57 PCG eyes who had final visit IOP record, 78.9% of them had successful IOP control (complete 64.9% and qualified 14.0%). While IOP > 21 mmHg was present in 21.1% of the failed eyes.

For JOAG, 91.3% (21/23 eyes) had successful control (complete 21.7% and qualified 69.6%). For eyes with secondary glaucoma, 83.9% (25/29) had successful IOP control (complete 3.2% and qualified 80.6%). While all of glaucoma suspect eyes had a successful IOP control (complete 73.7% and qualified 26.3%) (Table 7).

**Table 7:** Intraocular Pressure Outcomes (Minimum 6-Month Follow-Up)

	**Childhood Glaucoma**	**PCG**	**JOAG**	**Secondary Glaucoma**	**Glaucoma Suspect**
No. of eyes n (%)	130	57	23	31	19
Complete success	57 (43.8%)	37 (64.9%)	5 (21.7%)	1 (3.2%)	14 (73.7%)
Qualified success	54 (41.5%)	8 (14.0%)	16 (69.6%)	25 (80.6%)	5 (26.3%)
Complete or qualified Success	111 (85.4%)	45 (78.9%)	21 (91.3%)	26 (83.9%)	19 (100.0%)
Failure	19 (14.6%)	12 (21.1%)	2 (8.7%)	5 (16.1%)	-

*JOAG* Juvenile Open-Angle Glaucoma, *PCG* Primary Congenital Glaucoma

Success was defined numerically as an IOP ≤ 21 mmHg without (complete success) or with (qualified success) medication

With regard to IOP reduction, for all eyes with IOP records at final follow-up visit, mean IOP reduced from 37.1 mmHg at baseline to 16.8 mmHg (mean IOP reduction = 20.3 mmHg; 95% CI, 17.9–22.7; *P* < 0.001, paired *t* test). The PCG eyes had a mean IOP reduction of 24.5 mmHg (95% CI, 20.7–28.2, *P* < 0.001, paired *t* test), eyes with JOAG had a mean IOP reduction of 25.7 mmHg (95% CI, 20.0–31.3; *P* < 0.001, paired *t* test), and eyes with secondary glaucoma had a mean IOP reduction of 17.1 mmHg (95% CI, 17.0–34.1; *P* < 0.001, paired *t* test) (Table 5).

## Discussion

Childhood glaucoma is a vision-threatening disease characterized by elevated intraocular pressure (IOP)-related damage to the eye caused by a variety of conditions. IOP control is critical for optimal visual outcomes and can be achieved through both medical and surgical therapy. This study analyzed the profile of childhood glaucoma among patients presented to the Jimma Medical Center from September 2019 to August 2022.

During the 3-year study period PCG was the most common type of childhood glaucoma (42%), consistent with previous studies [11–14]. The mean age at presentation of PCG patients was 2.7 years old, which is significantly older than the mean age at presentation of 11 months in developed countries [15], however similar to reports in developing countries [16, 17]. Late presentation of PCG patients in the developing world is a well-known challenge [18]. Many factors contribute to this, including limited access to healthcare due to socioeconomic status, healthcare costs, physical distances required to travel to healthcare centers, and potential financial impact on parents' livelihoods.

In this study, only ≈ 3% of the patients had family history of childhood-onset glaucoma and, for most patients’ the history of consanguinity was unknown. This finding is smaller than a 20% positive family history of childhood-onset glaucoma observed in a retrospective study in USA [5]. This low prevalence is due to lack of proper detection and documentation.

Majority (94.4%) of PCG eyes in this study received glaucoma medications before surgery. Greater than a report from a multicenter, prospective study, where 57% of PCG eyes received antiglaucoma medication before surgery [19]. This is due to higher IOP at presentation in this study, increasing the use of antiglaucoma medications as temporizing measure before surgery. In this multicenter prospective study, topical CAI was used in 82.2%, topical beta-blocker in 50% and oral CAI in 2.7% of PCG eyes. In the current study, topical beta-blockers (100%) and systemic carbonic anhydrase inhibitors (76.4%) were the most frequent medications prescribed. Systemic CAIs prescribed in higher percentage of PCG eyes in comparison to this multicenter, prospective study [19], and in a retrospective study in Egypt [17]. This highlights how crucial it is to utilize drugs correctly while being mindful of any specific side effects they may have in children.

PCG is considered a surgical condition, and angle surgery is the first-line procedure, either as goniotomy, trabeculotomy with a trabeculotome probe, or an illuminated microcatheter or other filament to achieve 360-degree incisions [20]. However, primary CTT for PCG has been recommended in some patients who appear late and have a more severe phenotype [21–23] because it has a higher success rate for IOP control than trabeculotomy or trabeculectomy alone.

In this study, 93.4% of PCG eyes treated surgically. Majority of them underwent primary CTT as first line surgery, followed by trabeculectomy and goniotomy. Late presentation has been contributed to CTT being as a favored procedure. This finding is consistent with other reports in developing countries [16, 17, 24]. In centers in the United States, United Kingdom, Germany, Saudi Arabia, Singapore, and Israel, angle surgery alone was the first line, according to a report by Papadopoulos et al. [19].

In our study, about 22.4% of PCG eyes required medical treatment after surgery, with 1.24 mean number of antiglaucoma drugs (AGDs). This finding is smaller than the observed finding in a retrospective study in Egypt, 49.5% of PCG eyes required medical treatment after surgery, with 1.7 mean number of AGDs [17]. This signifies the higher surgical success rate of CTT in our center.

The mean final visit IOP of 17 ± 7.7 mmHg observed in our study is comparable to the mean final visit IOP of 16.2 ± 7.3 mmHg observed in a multicenter, prospective study of patients with PCG [19]. Additionally, it is comparable to the mean final visit IOP reported in a retrospective study from Egypt, which was 20.60 ± 3.03 mmHg [17].

With regard to success of childhood glaucoma management, considering VA outcome is more important. Achieving the goal of preserving visual function in children with glaucoma is influenced by factors other than IOP, such as corneal clarity, refractive status, and amblyopia, which also must be addressed. There was a statistically significant a mean logMAR VA deterioration of -0.26, (95% CI, -0.5—-0.02; *p* = 0.033, paired *t* test) in quantitative VA in PCG (albeit small number of eyes). Furthermore, the final quantitative VA was reported with refractive correction in only 5.3% of PCG eyes. The importance of ametropic correction (and amblyopia treatment) cannot be stressed enough if the child is to achieve his/her maximum visual potential. This is not to be underestimated, because better VA is associated with both higher functional visual ability and quality of life [25].

With regard to control of IOP ≤ 21 mmHg, there was a high degree of overall success for PCG (78.9%), with more complete than qualified success. Overall success for JOAG was 90.5% and secondary glaucoma was 86.2%, with more qualified than complete success, likely reflecting the greater use of medication as first-line therapy in these groups as opposed to surgery. This finding is similar to 72.9% overall success for PCG, with more complete than qualified success and, 83.7% overall success for JOAG and 76% for secondary glaucoma, with more qualified than complete success in a multicenter, prospective study [19].

Accurately assessing the prevalence of family history of childhood-onset glaucoma and consanguinity in patients with childhood glaucoma was difficult because of the lack of documentation. Another limitation of the study was being a single center report.

In conclusion, in this retrospective study of children < 18 years of age with glaucoma, the most common glaucoma was PCG and CTT was the most common surgery performed in these eyes. Steroid-induced glaucoma was the most common type of secondary type of secondary glaucoma; appropriate measures should be taken to avert this preventable glaucoma. One of a well-recognized challenge in developing countries, late presentation of patients with PCG, was also observed in our study. Which highlights, the need of increasing access to eye-care service and awareness of childhood glaucoma as a major public health issue. In addition, this study encourages the establishment of prospective studies and surgical trials in this field.

## Data Availability

The datasets used and/or analyzed during the current study available from the corresponding author on reasonable request.

## Abbreviations

AGD: Antiglaucoma drugs
CAI: Carbonic anhydrase inhibitor
CCT: Combined trabeculotomy–trabeculectomy
CGRN: Childhood Glaucoma Research Network Classification
CPC: Cyclophotocoagulation
EUA: Examination Under Anesthesia
GDD: Glaucoma Drainage Device
IOP: Intraocular Pressure
JIA: Juvenile Idiopathic Arthritis
JMC: Jimma Medical Center
JOAG: Juvenile Open-Angle Glaucoma
logMAR: Logarithm of minimum angle of resolution
SD: Standard Deviation
VA: Visual Acuity
